# Targeting reactivated toxoplasmosis: therapeutic efficacy of the green-synthesized copper nanoparticles combined with pyrimethamine

**DOI:** 10.1128/aac.00019-26

**Published:** 2026-06-04

**Authors:** Sultan F. Alnomasy, Aishah Alatawi

**Affiliations:** 1 Department of Medical Laboratory Sciences, College of Applied Medical Sciences, Shaqra University 248368, Shaqra, Saudi Arabia; 2 Department of Biology, Faculty of Science, University of Tabuk 125900, Tabuk, Saudi Arabia; The Children's Hospital of Philadelphia, Philadelphia, Pennsylvania, USA

**Keywords:** toxoplasmosis, nanomedicine, immunosuppression, treatment

## Abstract

This investigation seeks to determine their potential efficacy in reducing parasite load and mitigating disease progression in immunocompromised hosts. The green synthesis of copper nanoparticles (CNPs) was performed using an extract from *Rumex vesicarius*. The chronic toxoplasmosis model was developed using the ME49 strain of *Toxoplasma gondii*. RT in the mice was achieved using dexamethasone (0.25 mg/kg) for 30 days. Then, mice were randomly assigned to 10 distinct groups, which were orally treated with CNP alone (10 and 20 mg/kg) and in combination with pyrimethamine (PM, 5 mg/kg) for 28 days. Next, parasite burden, spleen cell proliferation, and cytokine analysis, molecular analysis of inflammatory and apoptosis gene expression, oxidative/antioxidative biomarkers, and biochemical analysis were evaluated. CNP exhibits a uniform distribution and spherical morphology with an average diameter of 40 nm. CNP, mainly in combination with PM, significantly enhanced the survival rate in RT mice (*P* < 0.001), whereas it markedly yielded the most substantial reduction in parasite burden across all organs assessed (*P* < 0.001). The CNP 20 mg/kg + PM 5 mg/kg group demonstrated the highest increases in the gene expression of immune factors (3.78- to 5.79-fold change) (*P* < 0.001), whereas it reduced the expression of pro-apoptotic markers (*P* < 0.01). CNP + PM indicated a synergistic interaction in mitigating liver and kidney damage associated with reactivated toxoplasmosis. The combined administration of CNP and PM significantly decreased the parasitic load in cases of reactivated toxoplasmosis, concurrently augmenting antioxidant capacity and innate immune function. These results indicate that CNP holds potential as a valuable adjunctive therapy to enhance treatment efficacy in reactivated toxoplasmosis.

## INTRODUCTION

The infectious disease toxoplasmosis is caused by a parasite, *Toxoplasma gondii*, which is an obligate intracellular protozoan that can infect almost any animal, including humans (1). Most healthy people will have no significant illness from a *Toxoplasma* infection or only experience mild, self-limiting symptoms like temporary lymph node swelling. However, there can be rare cases where an individual develops more severe infections, involving critical organs, even when no immune deficiency is present (2).

For patients with reduced cellular immunity, there is an increased likelihood of reactivation of latent *T. gondii* infections (referred to as reactivated toxoplasmosis) (3). *T. gondii* infects hosts on a cellular level, and after primary infection, the parasite remains dormant in host tissue as bradyzoite-containing cysts, with the brain, eyes, and muscles being the major areas of infection. Patients who have conditions such as HIV/AIDS, receive organ transplantations, have malignancies, or receive immunosuppressive therapies are susceptible to ruptured bradyzoite-containing cysts (from these conditions) releasing tachyzoites, resulting in an active disease state (4). In terms of clinical presentation, patients with reactivated toxoplasmosis most frequently manifest with cerebral toxoplasmosis, with signs and symptoms of focal neurological deficits, seizures, and altered mental status, although there are also ocular and disseminated presentations of the disease. The continued high morbidity and mortality from reactivated toxoplasmosis in patients with compromised immune systems have highlighted the need for early diagnosis and timely antiparasitic treatment (3, 4).

Current standard therapy relies primarily on the synergistic combination of pyrimethamine and sulfadiazine, with the co-administration of folinic acid to mitigate hematologic toxicity (5). If the combined regimen of pyrimethamine and sulfadiazine is not tolerated or can be contraindicated, there are some alternatives, which include spiramycin (especially used in pregnant women), as well as clindamycin or minocycline. However, the ability of these medications to effectively treat *T. gondii* has been limited by adverse event profiles (such as bone marrow suppression, allergic reactions, gastrointestinal problems/side effects; or teratogenicity associated with the use of these agents, in particular, during the early stages of gestation) and some strains of *T. gondii* exhibiting decreased susceptibility to the more commonly used antiparasitic drugs (6). Thus, although drug discovery is continuing to make progress, to date, there has not been a universally safe and effective method to treat all individuals with toxoplasmosis, particularly for its active and latent forms (5, 6).

Nanomedicine provides an innovative therapy for parasitic infections, specifically toxoplasmosis, through improved delivery of drugs directly into the infected cells containing the parasites, improved absorption of these drugs in the host, and a reduction in the toxicity associated with the drugs administered systemically. Nanoproducts have demonstrated potential to enhance the efficacy of the available antiparasitic agents, as well as to address the limitations of the currently used therapies (7). Nanoscale copper particles (copper nanoparticles [CNPs]) have distinct physical and chemical characteristics, such as their large amount of exposed area, chemical activity, and ability to make chemical species known as reactive oxygen species (8). Due to these characteristics, CNPs are useful for multiple medical uses, including the treatment of infections caused by bacteria, viruses, and cancer, as well as how they help with delivering drugs to specific places in the body and healing wounds (8, 9). CNPs also provide effective treatments against parasitic infections, including *T. gondii,* by affecting and breaking down the structure of the membrane surrounding the parasite cells, causing unwanted oxidative conditions and preventing the parasite from growing inside the cell (10). Their small size makes it easier for cells to absorb them and for doctors to deliver specific treatments. The two main ways of creating or making CNP today are using environmentally friendly methods or using natural substances (8). The environmentally friendly, or “green,” methods use plant materials, bacterial products, or other natural materials to make CNP (11). These environmentally friendly methods do not use harmful chemicals, do not harm the environment, and create biocompatible particles.

Numerous studies have shown the effectiveness of CNP in combating *T. gondii*; however, the majority of these studies have only tested the use of CNP on acute and chronic infections or in an *in vitro* environment (12–14). In addition, no studies to date have specifically evaluated the effects of CNP on reactivated toxoplasmosis, which occurs under immunosuppressive conditions and represents a clinically significant challenge. Consequently, more research is necessary to determine whether CNPs provide both an effective and safe treatment option for reactivated infections, particularly in immunocompromised individuals. *Rumex vesicarius* L., a member of the Polygonaceae family, is a medicinal plant widely distributed in arid and semi-arid regions and has been traditionally used in folk medicine for the treatment of several inflammatory and infectious conditions. Phytochemical investigations have revealed that this plant is a rich source of biologically active secondary metabolites, including flavonoids, phenolic acids, anthraquinones, tannins, and other polyphenolic compounds. These phytoconstituents possess strong antioxidant and redox-active properties that enable them to act as natural reducing and stabilizing agents during the green synthesis of metallic nanoparticles. In plant-mediated nanoparticle synthesis, such polyphenolic compounds facilitate the reduction of metal ions while functioning as capping agents that improve nanoparticle stability and biological compatibility. Moreover, extracts of *R. vesicarius* have been reported to exhibit notable antioxidant, antimicrobial, and anti-inflammatory activities, suggesting the presence of bioactive molecules that may contribute to the enhanced biological performance of the synthesized nanomaterials. Considering these properties, *R. vesicarius* extract was selected in the present study as a natural and eco-friendly reducing and capping agent for the green synthesis of CNP.

Although green synthesis of CNP using plant extracts has been widely explored, studies investigating the therapeutic potential of plant-mediated CNP in experimental models of parasitic infections remain very limited. In particular, the potential application of such nanoparticles for the treatment of reactivated toxoplasmosis, a severe and life-threatening condition in immunocompromised hosts, has not been sufficiently investigated. Therefore, the present study aimed to synthesize CNP using *R. vesicarius* extract and to evaluate their antiparasitic efficacy, potential synergistic interaction with pyrimethamine, and associated immuno-oxidative responses in a murine model of reactivated toxoplasmosis.

## MATERIALS AND METHODS

### Materials

Copper sulfate (CuSO4, >99% purity) and QN were purchased from Sigma-Aldrich, Germany. Fetal bovine serum (FBS), ketamine, and xylazine were purchased from Merck, Germany. The other chemicals used in this research study were of the highest purity.

### Green synthesis of CNP

Leaves of *Rumex vesicarius* L. were collected from an herbal market in Al Duwadimi County, Saudi Arabia, and identified by the Faculty of Biological Sciences and Humanities, Shaqra University. The leaves were washed, air-dried, and powdered. The hydroalcoholic extract was produced by extracting 10 g of dried powder with 100 mL of 50% ethanol solution in a water bath set at 45°C for a total of 8 hours, filtering, and drying at 50°C, as described thoroughly elsewhere (15). Green synthesis of CNP was carried out using the hydroalcoholic extract of *R. vesicarius* as a reducer and capping agent, following the previously reported procedure (16), with some modifications. In short, an equal volume of the aqueous solution of 50 mL of hydroalcoholic extracts of *R. vesicarius* was mixed with 50 mL of a 3 mM solution of copper sulfate⋅5H_2_O (Sigma-Aldrich, Germany), then stirred for 12 hours in a water bath set at a temperature of 65°C, during which time the color of the solution turned from blue to brownish red, signaling the production of CNs, which was optimized using initial color changes.

#### Physicochemical properties

UV-visible absorption spectrum analysis was used to verify the formation of GSCNPs. This experiment was conducted using a UV-visible spectrophotometer from Jenway laboratory instruments (model number 6505) in the UK. The biomaterial nanoparticles obtained from the procedure were separated through cold centrifugation at 1,000 rpm and a temperature of 4°C for a period of 15 minutes. The structural and physicochemical characteristics of the CNP were assessed through a combination of analytical methods. The functional groups that take part in the formation of the nanoparticles were determined through Fourier-transform infrared spectroscopy analysis (FTIR; Bruker Optics, Billerica, MA). The surface morphologies, sizes, and compositions of the nanoparticles were determined through field emission scanning electron microscopy analysis, energy-dispersive X-ray spectroscopy (FE-SEM/EDX; TESCAN MIRA3).

### Animals

One hundred male Balb/c mice, aged 6–8 weeks, weighing 25–30 grams, were collected from the Animal House, Imam Abdurrahman Bin Faisal University, Saudi Arabia. The animals were kept in standardized environments, including temperatures of 24°C ± 1°C, 12 hours of light and darkness, and relative humidity ranging from 40% to 70%. Additionally, the animals were allowed free access to food and water throughout the experiment period.

### Induction of infection and establishment of chronic toxoplasmosis

ME49 strain of *T. gondii* was procured from Shaqra University, Saudi Arabia, and subsequently maintained through serial intraperitoneal passages in BALB/c mice. The chronic toxoplasmosis model was developed using the ME49 strain of *T. gondii*. Each mouse was injected intraperitoneally with 0.5 mL homogenate suspension from the brain tissue of an infected donor mouse, with a concentration of 20–25 cysts, together with a penicillin-streptomycin mixture of antibiotics (12).

### Induction of RA in the animal model of toxoplasmosis

On the 28th day post-infection, reactivation in the mice was achieved using dexamethasone (Sigma Aldrich, Germany), which was given to the mice via the oral route at a dose of 10 mg/kg body weight daily for a period of 28 days (17).

### Study design and grouping

The mice were randomly assigned to 10 distinct groups, each comprising 10 individuals, including the following (16):

(i) Healthy mice + normal saline (HMNC)
(ii) Healthy mice + CNP 10 mg/kg (HM + CNP10)
(iii) Healthy mice + CNP 20 mg/kg (HM + CNP20)
(iv) Non-RA-infected mice (NRAI)
(v) RA-infected mice + Normal saline (RA + NC)
(vi) RA-infected mice + PM 10 mg/kg (RA + PM 10)
(vii) RA-infected mice + CNP 10 mg/kg (RA + CNP 10)
(viii) RA-infected mice + CNP 20 mg/kg (RA + CNP 20)
(ix) RA-infected mice + CNP 10 mg/kg + PM 5 mg/kg (RA + CNP 10+ PM5)
(x) RA-infected mice + CNP 20 mg/kg + PM 5 mg/kg (RA +CNP 20+ PM5)

Treatment regimens for mice in groups IV through X commenced on the 28th day post-infection and were maintained for a period of 28 days. Additionally, the survival rates and body weights of the experimental subjects were systematically monitored via daily evaluations throughout the study duration. Ten mice were included in each experimental group, which is consistent with sample sizes commonly used in murine models of toxoplasmosis reported in previous studies. All animals were included in the final analyses, and no exclusions were made during the study. Samples obtained from the same animals were used for the different experimental assays, including parasite burden assessment, cytokine measurements, oxidative stress biomarker analysis, and gene expression evaluation, to minimize the number of animals used while maintaining experimental consistency.

#### Sample collection

At 8 weeks post-infection, surviving mice from groups IV through X (*n* = 6 per group) were anesthetized via intraperitoneal administration of ketamine (15 mg/kg) combined with xylazine (100 mg/kg). Following anesthesia, the entire brain, heart, liver, kidney, and spleen tissues were aseptically collected for further analysis. Additionally, blood samples of the tested mice were obtained via cardiac puncture and transferred into sterile tubes containing anticoagulant to facilitate serum isolation.

#### Spleen cell proliferation assay and cytokine analysis

*In vitro* spleen cell proliferation was analyzed by extracting spleen cells from the mice under sterile conditions from the spleens of the mice involved in the research. The cells were then washed with phosphate-buffered saline (PBS). Cells were seeded at a concentration of 1 × 10^6^ cells/mL with RPMI-1640 media supplemented with 10% fetal calf serum (Merck, Germany). Stimulation was carried out with Con A (Sigma-Aldrich, Germany) at a concentration of 5 μg/mL in 96-well plates. After a 72-h incubation period, cell proliferation was analyzed for 3-(4,5-Dimethylthiazol-2-yl)−2,5-diphenyl tetrazolium bromide assay (Sigma-Aldrich, Germany) based on the method described previously (18). Subsequent cytokine analysis in the supernatant was followed as before, with cytokine production of IL-2, IFN-γ, and tumor necrosis factor alpha (TNF-α) being quantified with the Mouse ELISA kits (Abcam, USA) of ab46096, ab282874, and ab208348, respectively.

#### Assessment of parasite burden by real-time PCR

The parasite load of *T. gondii* in various murine organs (brain, heart, liver, kidney, and blood) was quantified using reverse transcription-polymerase chain reaction (RT-PCR). Genomic DNA was isolated from tissue samples utilizing the Blood/Tissue DNA Extraction Kit (Qiagen, Germany) in accordance with the protocol provided by the manufacturer. For PCR amplification, the *B1* gene of *T. gondii* (GenBank, AF179871.1) was selected as the target, whereas the β-actin was also used as the internal control with primer sequences with primer sequences indicated in Table 1. (19, 20). The positive control consisted of total DNA extracted directly from RH-strain tachyzoites. PCRs were conducted in a 25 µL volume comprising 12.5 µL of 2× Taq PCR Master Mix, 2 µL each of forward and reverse primers, 5 µL of DNA template, and 3.5 µL of double-distilled water (ddH2O). Thermal cycling parameters included an initial denaturation at 94°C for 10 min, followed by 40 cycles of denaturation at 93°C for 30 seconds, annealing at 59°C for 30 seconds, and extension at 71°C for 30 seconds. Subsequently, RT-PCR amplification was performed in a 20 µL reaction mixture containing 10 µL SYBR Green Master Mix (Qiagen, Germany), 1 µL of each primer, 2 µL DNA template, and 6 µL ddH2O, employing identical cycling conditions as described for conventional PCR. The parasites in the samples were quantified using qPCR threshold cycle (CT) values in reference to the standard curve prepared using DNA from serial dilutions of RH-strain tachyzoites. Data are shown as tachyzoite equivalents per milliliter of blood or milligram of tissue (21).

**TABLE 1:** The sequence of the primers used for real-time PCR

Primers	Sequence (5′→3′)
TNF-α	F: TGAACTTCGGGGTGATCGGT R: GGTGGTTTGTGAGTGTGAGGG
NF-κB	F: GCGGGAGAGGGGATTCCCTGCGGCCCCG R: CGGGGCCGCAGGGAATCCCCTCTCCCGC
TLR4	F: AGCTTTGGTCAGTTGGCTCT R: CAGGATGACACCATTGAAGC
Caspase-3	F: TTCATTATTCAGGCCTGCCGAGG R: TTCTGACAGGCCATGTCATCCTCA
Bax	F: GGCTGGACACTGGACTTCCT R: GGTGAGGACTCCAGCCACAA
Bcl-2	F: CATGCCAAGAGGGAAACACCAGAA R: GTGCTTTGCATTCTTGGA TGAGGG
β-actin	F: GTGACGTTGACATCCGTAAAGA R: GCCGGACTCATCGTACTCC

### Molecular analysis of inflammatory and apoptosis gene expression

#### RNA extraction

Total RNA isolation was done using the Qiagen RNA extraction kit (Qiagen, Germany) according to the manufacturer’s guidelines. To eliminate any genomic DNA, DNase I (Qiagen, Germany) was used on the isolated RNA preparations. Integrity testing of isolated RNA was done by electrophoresis using a 1.5% agarose gel along with 2% formaldehyde.

#### cDNA synthesis

The concentrations of the RNAs were determined using a NanoDrop spectrophotometer (Thermo Fisher Scientific, USA). Then, the synthesis of cDNA was conducted using the cDNA synthesis kit from Qiagen GmbH (Germany).

#### Primers

Primer sequences for *TNF-α*, *NF-κB p65*, *TLR4*, *Caspase-3, BAX, Bcl-2*, and *β-actin* gene sequences, respectively, are shown in Table 1. These primers had been verified using the National Center for Biotechnology Information (NCBI) gene database, and the results had been verified using the Blast search tool.

#### Real-time PCR

RT-PCRs were optimized to a total volume of 20 μL, consisting of 10 μL of SYBR Green Master Mix (Qiagen, Germany), 1 μL of each of the forward and reverse primers (10 μM), 1 μL of cDNA, and nuclease-free water to fill the remaining volume. The thermal cycling profile involved an initial denaturation step of 8 minutes at 97°C, followed by 40 cycles of amplification, which cycled between denaturation for 10 seconds at 97°C and annealing and extension for 30 seconds at 65°C. Calculation of the quantitative cycles threshold (Ct) values was done by the 2^−ΔΔCt^ formula and evaluated by Bio-Rad IQ5 Optical System Software (USA) (16).

### Impact on oxidative and antioxidative biomarkers

Serum levels of the tested mice were prepared to facilitate the evaluation of oxidative and antioxidative biomarkers. Quantitative analyses of malondialdehyde (MDA), glutathione peroxidase (GPx) activity, and superoxide dismutase (SOD) enzymatic function were conducted utilizing commercially available assay kits from Abcam, USA (MDA: Abcam, Cat. No. 118970; SOD: Abcam, Cat. No. ab65354; GPx: Abcam, Cat. No. ab65354), following the protocols prescribed by the manufacturer (12).

### Biochemical analysis for safety evaluation

The obtained serum samples were analyzed using commercially available assay kits procured from Roche, Germany. These assays were employed to quantify renal biomarkers, specifically blood urea nitrogen (BUN) and creatinine (Cr), as well as hepatic biomarkers, including alanine aminotransferase (ALT) and aspartate aminotransferase (AST) (22).

### Statistical analysis

All statistical analyses were performed using SPSS software version 26.0. Data were expressed as mean ± standard deviation (SD). Differences among experimental groups were analyzed using one-way analysis of variance (ANOVA). When a significant difference was detected, Tukey’s multiple comparison post hoc test was applied to evaluate pairwise differences between groups and to control for Type I error associated with multiple comparisons. Each experimental group consisted of ten animals (*n* = 10). A *P*-value < 0.05 was considered statistically significant.

## RESULTS AND DISCUSSION

### SEM analysis of CNP

Analysis revealed that CNP exhibited a uniform distribution, predominantly spherical morphology, and smooth surface texture. The micrographs presented in Fig. S1 demonstrate that the majority of the particles fall within a narrow nanometer size range, with an average diameter of approximately 40 nm. The attainment of such a size suggests that the bioactive constituents within the plant extract effectively regulated the nucleation and growth processes of the nanoparticles. From an application standpoint, this morphological characteristic is particularly significant, as smaller and uniformly sized nanoparticles typically display enhanced physicochemical stability and more efficient interactions with biological systems (23).

### XRD analysis of CNP

XRD spectroscopy was conducted to determine the elemental composition of the nanoparticles, confirming copper as the predominant element, with no notable signals indicative of impurities. This outcome underscores the high purity of the synthesized nanoparticles and attests to the efficacy of the employed green synthesis approach. These findings are consistent with numerous studies reporting the biosynthesis of CNP using various plant extracts. Notably, in contrast to many prior investigations where particle sizes exhibited a broader distribution, often exceeding 100 nm, the comparatively smaller size observed in the present study may confer a considerable biological advantage. Existing literature indicates that biogenic nanoparticles with dimensions below 100 nm demonstrate enhanced biological activities, including antimicrobial and antiparasitic effects, attributable to their increased surface-to-volume ratio (24) (Fig. S1).

### FTIR analysis of CNP

FTIR spectrum provided critical insights into the chemical composition of the nanoparticle surface coating (Fig. S2). A broad absorption band centered around 3,449 cm⁻¹ was assigned to hydroxyl groups, indicating the involvement of phenolic and polyphenolic compounds from the plant extract in both the reduction and stabilization of the nanoparticles. Peaks observed near 2,926 cm⁻¹ correspond to C–H stretching vibrations, suggesting the presence of aliphatic compounds from the plant contributing to the surface capping of the particles. Additionally, the absorption band at 1,659 cm⁻¹ is attributed to C=O stretching vibrations or conjugated aromatic structures, which have been previously identified as crucial factors in the stabilization of metal nanoparticles (3). The spectral features around 1,362 cm⁻¹ likely arise from carboxylic or methylene groups, which may influence the surface charge and thereby inhibit nanoparticle aggregation. Notably, a distinct peak observed at approximately 504 cm⁻¹ is characteristic of Cu–O bond vibrations, confirming the successful synthesis of copper oxide nanoparticles. This finding implies that secondary metabolites present in the plant extract function not only as reducing agents but also play a structural role in defining the final nanoparticle architecture (19).

### UV–Vis analysis of CNP

UV–Vis spectroscopy further substantiated the formation of nanoparticles. The absorption spectrum exhibited two prominent maxima at 273 nm and 375 nm (Fig. S3). The peak at the shorter wavelength is attributed to organic compounds from the plant extract adsorbed onto the nanoparticle surface, whereas the absorption band within the 300–400 nm range corresponds to SPR. This phenomenon, typically observed in metal and metal oxide nanoparticles of nanoscale dimensions, is highly sensitive to particle size and morphology. The detection of SPR within this spectral window confirms that the synthesized nanoparticles possess dimensions conducive to quantum confinement effects. These findings align with recent reports documenting UV–Vis absorption peaks for biosynthesized CNP between 300 and 350 nm, which have been linked to enhanced bioactivity (20). Taken together, the structural and optical characteristics of the CNP suggest their potential utility in antiparasitic and biomedical applications.

### Survival rate

**Fig 1:**
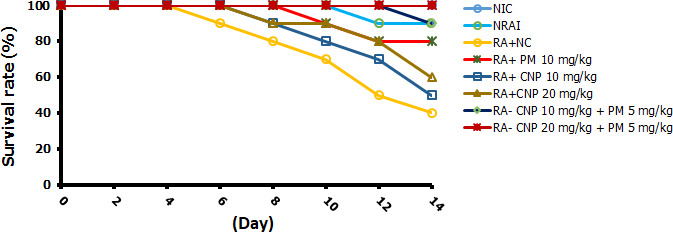
Survival rate of the mice with reactivated toxoplasmosis (RA) after 28 days of treatment with green synthesized CNPs alone and in combination with PM. Statistical analysis was conducted utilizing one-way ANOVA, accompanied by Tukey’s post hoc test. The results are presented as mean ± standard deviation, with a sample size of eight mice per group (*N* = 10 mice).

Statistical analysis revealed a significant difference in survival rates among the groups over the 14-day follow-up period (Fig. 1). Administration of CNP demonstrated a dose-dependent protective effect, with survival rates of approximately 50% at 10 mg/kg and 60% at 20 mg/kg (*P* < 0.05). The highest survival rates were observed in the combination treatment groups. Specifically, the RA + CNP 10 mg/kg + PM 5 mg/kg group exhibited survival rates of approximately 90% by the study’s conclusion, whereas the RA + CNP 20 mg/kg + PM 5 mg/kg group experienced no mortality, maintaining 100% survival throughout the entire duration. Comparative analysis of survival curves indicated that combination therapy significantly enhanced survival relative to monotherapy (*P* < 0.001).

### Spleen cell proliferation

**Fig 2:**
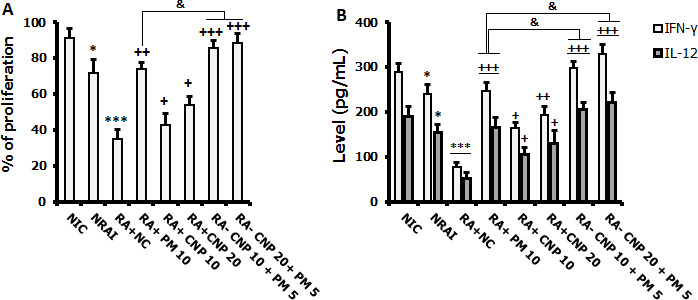
*In vitro* effect of the green synthesized CNPs alone and in combination with PM on the spleen cell proliferation (**A**) and cytokine analysis (**B**). The mice with RA toxoplasmosis after 28 days of treatment. Statistical analysis was conducted utilizing one-way ANOVA, accompanied by Tukey’s post-hoc test. The results are presented as mean ± standard deviation, with a sample size of eight mice per group (*N* = 3). Non-infected control mice (NIC); non-RA-infected mice (NRAI); RA-infected mice + normal saline (RA + NC); RA-infected mice + PM 10 mg/kg (RA + PM 10); RA-infected mice + CNP 10 mg/kg (RA + CNP 10); RA-infected mice + CNP 20 mg/kg (RA + CNP 20); RA-infected mice + CNP 10 mg/kg + PM 5 mg/kg (RA + CNP 10+ PM5); RA-infected mice + CNP 20 mg/kg + PM 5 mg/kg (RA + CNP 20+ PM5). *P < 0.05 and ***P < 0.001 significant difference compared to NIC; + *P* < 0.05, ++ *P* < 0.01, and +++ *P* < 0.001 compared to the RA + NC. & *P* < 0.05 compared to the RA + PM10.

The impact of CNP on Con A-stimulated proliferation of mice spleen lymphocytes was evaluated using the MTT assay. The findings demonstrated that CNP, mainly in combination with PM, significantly restored the proliferative capacity that had been suppressed during reactivation (*P* < 0.01). Notably, the highest proliferation rates in spleen cells were observed in mice treated with a combination of PM (5 mg/kg) and CNP at dosages of 10 and 20 mg, with no statistically significant difference compared to uninfected control mice (Fig. 2A).

### Spleen cell cytokines analysis

Cytokine profiles of splenocytes represent sensitive biomarkers in the context of preclinical drug assessment. For instance, in murine models of dexamethasone-induced reactivated toxoplasmosis, animals experiencing reactivation exhibit diminished production of IFN-γ and IL-10, alongside decreased populations of CD4^+^ and CD8^+^ T cells, relative to chronically infected controls. This pattern indicates suppression of Th1 responses and a concomitant shift toward a Th2-dominant immune profile following corticosteroid administration (17). Investigating the capacity of candidate therapeutics to restore or modulate these cytokine responses offers mechanistic insights into their immunomodulatory potential. Specifically, compounds that promote Th1 cytokine expression (such as IFN-γ and IL-12) or reestablish the Th1/Th2 equilibrium in splenocyte cultures may hold promise for enhancing host immune defenses and preventing parasite reactivation (17). The concentration of IFN-γ and IL-12 in splenocytes from the NIC and NRAI groups was within the upper physiological range, reflecting normal Th1 immune response functionality in healthy mice. Conversely, the RA + NC group exhibited a significant reduction in IFN-γ (76.3 pg/mL) and IL-12 (51.1 pg/mL) levels, indicative of cellular immune response suppression following RA induction (*P* < 0.001). Administration of CNP resulted in a dose-dependent elevation of IFN-γ, with the 20 mg/kg dose producing a more pronounced increase compared to the 10 mg/kg dose (*P* < 0.05). The combination treatment groups demonstrated the highest IFN-γ levels; specifically, the RA + CNP 10 + PM 5 mg/kg group showed IFN-γ concentrations approaching normal values (297.8 pg/mL), whereas the RA + CNP 20 + PM 5 mg/kg group exhibited IFN-γ levels (329.4 pg/mL) exceeding those of the control group, suggesting a robust enhancement of the Th1 response (*P* < 0.001). Treatment with CNP induced a dose-dependent increase in IL-12, with the 20 mg/kg dose exerting a stronger stimulatory effect than the lower dose. The combination therapy groups recorded the highest IL-12 levels; in the RA + CNP 10 + PM 5 (206.6 pg/mL), IL-12 levels approximated those of the normal group, and the RA + CNP 20 + PM 5 (221.7 pg/mL) group exhibited the peak IL-12 concentration (*P* < 0.001), demonstrating effective activation of the IL-12/IFN-γ axis (Fig. 2B).

### Parasite burden in the vital organs

**Fig 3:**
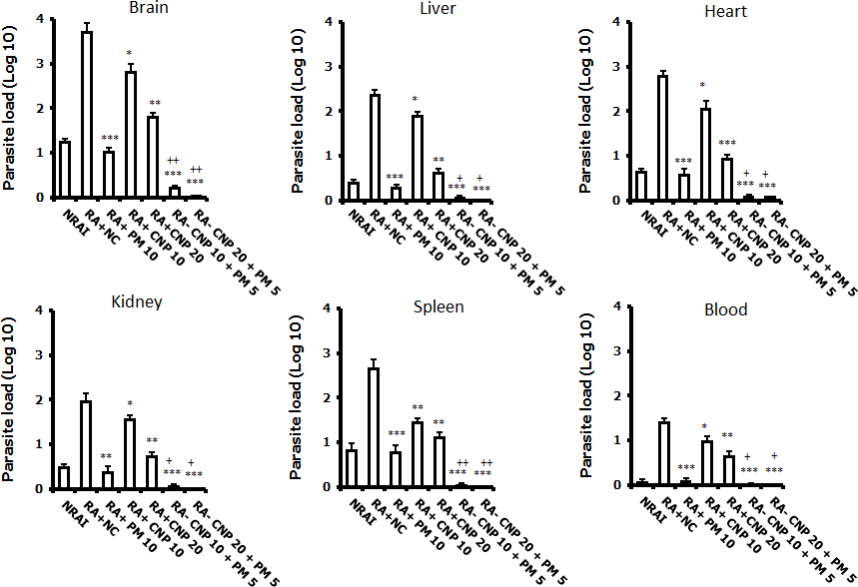
Parasite burden in the vital organs of the mice with RA toxoplasmosis after 28 days of treatment with the green synthesized CNPs alone and in combination with PM. Statistical analysis was conducted utilizing one-way ANOVA, accompanied by Tukey’s post hoc test. The results are presented as mean ± standard deviation, with a sample size of eight mice per group (*N* = 10 mice). Non-RA-infected mice (NRAI); RA-infected mice + Normal saline (RA + NC); RA-infected mice + PM 10 mg/kg (RA + PM 10); RA-infected mice + CNP 10 mg/kg (RA + CNP 10); RA-infected mice + CNP 20 mg/kg (RA + CNP 20); RA-infected mice + CNP 10 mg/kg + PM 5 mg/kg (RA + CNP 10+ PM5); RA-infected mice + CNP 20 mg/kg + PM 5 mg/kg (RA + CNP 20+ PM5). *P < 0.05 and ***P < 0.001 significant difference compared to RA + NC; + *P* < 0.05 and ++ *P* < 0.01 compared to the RA + PM10.

The analysis of parasite load across various organs revealed that the RA + NC group exhibited the highest parasite burden in all examined tissues, indicative of systemic infection dissemination (Fig. 3). Treatment with the single agent PM resulted in a significant reduction in parasite burden (*P* < 0.05). In contrast, administration of CNP demonstrated a dose-dependent decrease in parasite load, with the higher dose of 20 mg/kg producing a more pronounced effect than the lower dose (*P* < 0.01). Notably, combination therapy involving PM and CNP yielded the most substantial reduction in parasite burden across all organs assessed, including the brain, liver, heart, kidney, spleen, and blood. This effect was particularly evident in the RA + CNP 20 + PM 5 group, where parasite levels were significantly diminished relative to other groups and approached near-normal values (*P* < 0.001). These findings suggest a synergistic interaction between PM and CNP in mitigating systemic parasite dissemination, as well as a clear dose-response relationship for CNP efficacy.

Numerous *in vivo* investigations have substantiated the anti-toxoplasmic properties of CNP in murine models. For instance, Albalawi et al. (13) demonstrated that the administration of *Capparis spinosa* green-synthesized CNP (2 and 4 mg/kg) to BALB/c mice with chronic toxoplasmosis resulted in a significant reduction in both the number and size of *T. gondii* brain cysts. This therapeutic effect was markedly potentiated when combined with atovaquone 50 mg/kg. The observed decrease in parasitic load was concomitant with elevated levels of cellular immune markers, including IL-12, IFN-γ, and iNOS, indicative of activation of the host’s protective immune response. Similarly, Alanazi et al. (12) reported that oral delivery of *Lupinus arcticus*-synthesized CNP (5 and 10 mg/kg), mainly in combination with PYM at 5 mg/kg, elicited a dose-dependent diminution of brain cysts in *T. gondii*-infected mice. A synthesis of the current study’s findings alongside previous research indicates that the anti-toxoplasmic activity of CNP likely arises from the concurrent operation of multiple complementary mechanisms. First, the redox-sensitive properties of copper can disrupt the parasite’s oxidative homeostasis by elevating reactive oxygen species production, thereby inflicting damage on critical structures such as the cell membrane and mitochondria of *T. gondii*. This oxidative stress ultimately impairs the parasite’s survival and replication capacity (25). Second, CNPs contribute to creating an inhospitable environment for the intracellular parasite by augmenting the host’s cellular immune response, notably through the induction of pivotal cytokines, including IFN-γ and IL-12, as well as the activation of the inducible iNOS/NO pathway. The interplay between these direct antiparasitic effects and immune modulation may account for the pronounced reduction in parasite load observed across various animal models (25). Collectively, the alignment of the present study’s outcomes with data from three *in vivo* investigations suggests that green-synthesized CNP, particularly when employed adjunctively with conventional anti-toxoplasmosis treatments, holds considerable promise for managing *T. gondii* infections in chronic or reactivated states. Although variability in therapeutic efficacy among studies exists—potentially attributable to differences in nanoparticle synthesis methods, physicochemical characteristics, dosage, administration routes, or disease stage, the prevailing evidence supports the potential of CNP as a novel therapeutic avenue in toxoplasmosis management. Although the combined administration of CNP and pyrimethamine resulted in greater therapeutic efficacy compared with either treatment alone, the present study was not specifically designed to determine whether this interaction represents true pharmacological synergy. Formal synergy evaluation would require additional analyses, such as combination index or isobologram approaches. Nevertheless, the improved outcomes observed in the combination group suggest that CNP may enhance the antiparasitic activity of pyrimethamine through complementary mechanisms, including direct antiparasitic effects, modulation of host immune responses, and regulation of oxidative stress pathways. Further studies are warranted to more precisely characterize the pharmacological interaction between these agents.

### Analysis of inflammatory gene expression

In reactivated infections caused by *T. gondii*, the TLR4, NF-κB, and TNF-α signaling pathway are critically involved in the host immune defense (26). TLR4 detects the presence of the parasite and subsequently transmits signals to NF-κB, which functions as a transcription factor to induce the expression of genes associated with inflammation and immune defense, including TNF-α. TNF-α, in turn, activates macrophages and natural killer (NK) cells, promoting the production of ROS and NO, thereby inhibiting parasite replication. Disruption of this signaling cascade results in diminished immune responses and an increased parasitic load, whereas its proper activation facilitates effective infection control and preserves host tissue integrity (26, 27).

**Fig 4:**
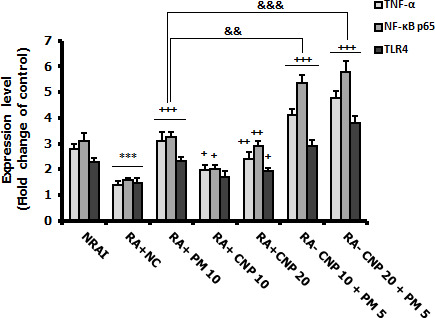
The expression level of TNF-α, NF-κB p65, and TLR4 in the mice with reactivated toxoplasmosis (RA) after 28 days of treatment with the green synthesized CNPs alone and in combination with PM. Statistical analysis was conducted utilizing one-way ANOVA, accompanied by Tukey’s post hoc test. The results are presented as mean ± standard deviation, with a sample size of eight mice per group (*N* = 10 mice). Non-RA-infected mice (NRAI); RA-infected mice + Normal saline (RA + NC); RA-infected mice + PM 10 mg/kg (RA + PM 10); RA-infected mice + CNP 10 mg/kg (RA + CNP 10); RA-infected mice + CNP 20 mg/kg (RA + CNP 20); RA-infected mice + CNP 10 mg/kg + PM 5 mg/kg (RA + CNP 10+ PM5); RA-infected mice + CNP 20 mg/kg + PM 5 mg/kg (RA + CNP 20+ PM5). ***P < 0.001 significant difference compared to NRAI; + *P* < 0.05, ++ *P* < 0.01, and +++ *P* < 0.001 compared to the RA + NC. && *P* < 0.01 and &&& *P* < 0.001 compared to the RA + PM10.

In the RA + NC group, representing mice with untreated reactivated toxoplasmosis, there was a significant reduction in the levels of inflammatory markers *TNF-α* (1.37-fold change), *NF-κB p65* (1.56-fold change), and *TLR4* (1.46-fold change) (*P* < 0.001). This decrease suggests a suppression of the host immune response and diminished activation of key inflammatory pathways during toxoplasmosis reactivation, potentially facilitating increased parasite burden and subsequent tissue damage. Administration of CNP alone exhibited dose-dependent effects. CNP at 10 mg/kg, a modest elevation in TNF-α (1.96-fold change), NF-κB p65 (2.01-fold change), and TLR4 (1.69-fold change) was observed, whereas at 20 mg/kg, these inflammatory markers increased significantly (TNF-α: 2.39-fold change, NF-κB p65: 2.89-fold change, TLR4: 1.94-fold change) relative to the RA + NC group (*P* < 0.05). These results indicate that CNP can potentiate the immune response and modulate host inflammation. The most pronounced enhancement of immune activation was noted in groups receiving combined treatment with CNP and PM. Specifically, the RA + CNP 10 + PM 5 group exhibited elevated levels of TNF-α (4.11-fold change), NF-κB p65 (5.34-fold change), and TLR4 (2.89-fold change), while the RA + CNP 20 + PM 5 group demonstrated the highest increases in these markers (TNF-α: 4.78-fold change, NF-κB p65: 5.79-fold change, TLR4: 3.78-fold change) (*P* < 0.001) (Fig. 4). This pattern suggests a synergistic interaction between CNP and PM in activating the TLR4/NF-κB signaling pathways, thereby amplifying the host immune response. The underlying mechanisms of CNP and combination therapy likely involve activation of the TLR4/NF-κB pathway, which promotes the production of TNF-α and other protective cytokines, contributing to parasite control. Additionally, these treatments enhance host immune defenses by inducing protective signaling and mitigating the immunosuppressive effects associated with toxoplasmosis reactivation. The synergistic effect observed with PM may further potentiate immune activation, improve therapeutic efficacy at reduced dosages, and minimize adverse effects. These findings align with prior studies demonstrating the capacity of CNP to modulate inflammatory and immune pathways (28). In line with our results, Albalawi et al. (13) reported that CNP synthesized via a green method using *C. spinosa* extract exhibited a high efficacy in preventing *T. gondii* infection in mice. This protective effect was associated with a significant upregulation of mRNA expression levels of IFN-γ, IL-12, and inducible iNOS in *T. gondii*-infected mice following a 2-week treatment with CNPs. Collectively, the data support the potential of CNP, particularly in combination with PM, as a promising strategy to augment immune responses and manage infection in cases of reactivated toxoplasmosis.

### Analysis of apoptosis gene expression

**Fig 5:**
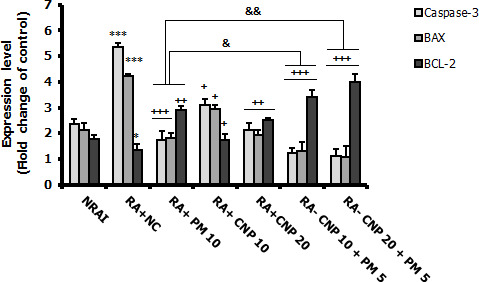
The expression level of Caspase-3, BAX, and Bcl-2 in the mice with RA toxoplasmosis after 28 days of treatment with the green synthesized CNPs alone and in combination with PM. Statistical analysis was conducted utilizing one-way ANOVA, accompanied by Tukey’s post hoc test. The results are presented as mean ± standard deviation, with a sample size of eight mice per group (*N* = 10 mice). Non-RA-infected mice (NRAI); RA-infected mice + Normal saline (RA + NC); RA-infected mice + PM 10 mg/kg (RA + PM 10); RA-infected mice + CNP 10 mg/kg (RA + CNP 10); RA-infected mice + CNP 20 mg/kg (RA + CNP 20); RA-infected mice + CNP 10 mg/kg + PM 5 mg/kg (RA + CNP 10+ PM5); RA-infected mice + CNP 20 mg/kg + PM 5 mg/kg (RA + CNP 20+ PM5). ***P < 0.001 significant difference compared to NRAI; + *P* < 0.05, ++ *P* < 0.01, and +++ *P* < 0.001 compared to the RA + NC. & *P* < 0.05 and && *P* < 0.01 compared to the RA + PM10.

The analysis of apoptosis indices revealed that in the RA + NC group, there was a significant upregulation of *Caspase-3* and *Bax* expression alongside a marked downregulation of *Bcl-2* expression compared to the NRAI group (*P* < 0.001). This expression profile suggests the activation of the mitochondrial-dependent apoptotic pathway during infection reactivation, concomitant with substantial cellular damage. Administration of CNP elicited a dose-dependent modulation of apoptotic signaling pathways. Specifically, treatment with 10 mg/kg CNP resulted in a modest reduction in *Caspase-3 and Bax* levels and a slight elevation in Bcl-2 expression (*P* < 0.05). In contrast, a higher dose of 20 mg/kg CNP produced a more pronounced decrease in pro-apoptotic markers and a significant increase in Bcl-2 relative to the RA + NC group (*P* < 0.01). The most pronounced cytoprotective effect was observed in groups receiving combined treatment with CNP and PM; both CNP doses administered alongside a low dose of PM led to a highly significant reduction in *Caspase-3* and *Bax* expression and a significant enhancement of *Bcl-2* levels compared to all monotherapy groups (*P* < 0.001) (Fig. 5). These findings demonstrate a potent synergistic interaction between CNP and PM in mitigating apoptosis induced by reactivated toxoplasmosis. Collectively, the data indicate that CNP, particularly at elevated doses and in combination with PM, effectively attenuates apoptosis and confers cellular protection during reactivated toxoplasmosis by modulating the Bax/Bcl-2 ratio and inhibiting Caspase-3 activation. This suggests that such combination therapy may represent a promising adjuvant strategy for therapeutic intervention. Numerous cellular studies have identified CNP as agents that induce apoptosis in cancer cells by generating excessive reactive oxygen species (ROS) and activating programmed cell death pathways (29, 30). However, the findings of the present study demonstrate that, within the context of reactivated toxoplasmosis infection and when appropriately formulated, CNP may serve as an adjunctive therapeutic modality by inhibiting proapoptotic pathways, evidenced by decreased expression of *Caspase-3* and *Bax* alongside increased levels of *Bcl-2,* and by mitigating oxidative stress. These results suggest that the apoptotic effects of CNP are contingent upon the specific biological environment, and that modulation of parameters such as dosage, biocompatible surface coatings, and tissue targeting can shift their impact from cytotoxic to cytoprotective.

### Oxidative and antioxidative biomarkers

**Fig 6:**
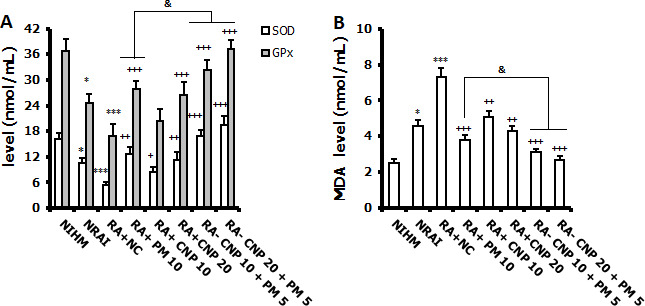
The serum level of (**A**) antioxidant enzymes of GPx and SOD as well as (**B**) malondialdehyde (MDA) in the mice with RA toxoplasmosis after 28 days of treatment with the green synthesized CNPs alone and in combination with PM. Statistical analysis was conducted utilizing one-way ANOVA, accompanied by Tukey’s post hoc test. The results are presented as mean ± standard deviation, with a sample size of eight mice per group (*N* = 10 mice). Healthy mice + normal saline (NIHM); non-RA-infected mice (NRAI); RA-infected mice + normal saline (RA + NC); RA-infected mice + PM 10 mg/kg (RA + PM 10); RA-infected mice + CNP 10 mg/kg (RA + CNP 10); RA-infected mice + CNP 20 mg/kg (RA+ CNP 20); RA-infected mice + CNP 10 mg/kg + PM 5 mg/kg (RA + CNP 10+ PM5); RA-infected mice + CNP 20 mg/kg + PM 5 mg/kg (RA + CNP 20+ PM5). *P < 0.05 and ***P < 0.001 significant difference compared to NIHM; + *P* < 0.05, ++ *P* < 0.01, and +++ *P* < 0.001 compared to the RA + NC. & *P* < 0.05 compared to the RA + PM10.

In healthy mice (NIHM), baseline values for SOD (16.1 U/mL), GPx (36.7 U/mL), and MDA (2.5 nmol/mL) were established to serve as reference points for comparison with other experimental groups (Fig. 6). In mice infected with *T. gondii* without reactivation (NRAI), a relative reduction in SOD (10.6 U/mL) and GPx (24.5 U/mL) levels was observed, accompanied by an elevation in MDA concentration (4.6 nmol/mL). These changes suggest the onset of oxidative stress and diminished antioxidant defenses induced by *Toxoplasma* infection, although the severity was less pronounced than in the reactivated toxoplasmosis group without treatment (RA + NS). In the untreated reactivated toxoplasmosis group (RN + NS), a more marked decline (*P*<0.001) in SOD (5.4 U/mL) and GPx (16.9 U/mL) was detected, alongside a significant increase in MDA (7.3 nmol/mL), indicating an exacerbated oxidative stress burden and enhanced lipid peroxidation relative to primary infection. This pattern implies that reactivation of toxoplasmosis results in substantial depletion of antioxidant reserves and increased membrane damage, with uncontrolled reactivation leading to tissue injury and cellular dysfunction. Among the treatment groups (PM, CNP, and their combination), differential effects were noted. CNP elicited a dose-dependent, modest modulation of these oxidative stress markers. The most pronounced (*P*<0.001) protective effect was observed with the combined treatment of CNP (20 mg/kg) and PM (5 mg/kg) in the reactivated toxoplasmosis model, where SOD (19.4 U/mL) and GPx (37.2 U/mL) activities exceeded normal baseline levels and MDA (2.7 nmol/mL) concentrations approximated those of healthy controls. These findings indicate that the combined therapeutic regimen exerts a synergistic effect, effectively restoring the host’s oxidative-antioxidant equilibrium and mitigating the oxidative damage associated with reactivated toxoplasmosis. CNPs function as antioxidant nanozymes, exhibiting enzymatic activities analogous to those of the endogenous enzymes SOD and GPx (31). These activities facilitate the neutralization of free radicals and the attenuation of lipid peroxidation, as indicated by reduced MDA levels (31). Consequently, CNPs contribute to the protection of cellular membranes, proteins, and DNA under conditions of oxidative stress. The underlying mechanisms may involve the activation of antioxidant signaling pathways, such as the Nrf2/HO-1 axis, the emulation of SOD and GPx enzymatic functions, and the regulation of ROS concentrations (32). The antioxidant efficacy of CNPs is significantly influenced by their physicochemical properties, including particle size, design, and surface coating, with smaller and appropriately coated nanoparticles demonstrating enhanced activity (33). In line with our results, Alanazi et al. (12) reported that the oral administration of *L. arcticus*-synthesized CNP at doses of 5 and 10 mg/kg, particularly when combined with PYM at 5 mg/kg, significantly modulated oxidative stress markers. Furthermore, CNP treatment alone markedly increased the levels of antioxidant factors in mice infected with *T. gondii*. These findings align with the results of the current study and provide a mechanistic rationale for the observed synergistic effect of combining CNPs with conventional therapeutic regimens, which collectively bolster antioxidant defenses and mitigate tissue damage associated with reactivated toxoplasmosis (12, 32).

Collectively, the therapeutic effects observed in the present study are likely mediated through multiple biological mechanisms. First, CNP may exert direct antiparasitic effects by disrupting parasite membranes, interfering with metabolic processes, and inducing intracellular oxidative damage in *T. gondii*. Second, the modulation of cytokine profiles suggests that CNP may enhance host immune responses, which play a critical role in controlling toxoplasmosis. Third, the observed improvement in antioxidant enzyme activities and reduction of lipid peroxidation markers indicate that CNP may contribute to restoring redox homeostasis in infected tissues. Nevertheless, this investigation is constrained by several limitations, including the lack of a comprehensive analysis of the underlying molecular signaling pathways, a restricted scope of histopathological evaluation, and the absence of pharmacokinetic profiling of the nanoparticles utilized.

### Biochemical analysis for safety evaluation

**Fig 7:**
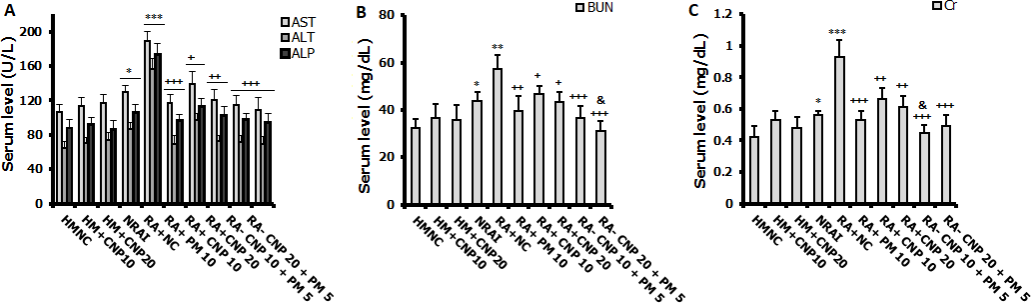
The serum level of (**A**) hepatic biomarkers, including alanine ALT and aspartate AST, and ALP, blood urea nitrogen (**B**) and creatinine (**C**) in the mice with RA toxoplasmosis after 28 days of treatment with the green synthesized CNPs alone and in combination with PM. Statistical analysis was conducted utilizing one-way ANOVA, accompanied by Tukey’s post hoc test. The results are presented as mean ± standard deviation, with a sample size of eight mice per group (*N* = 10 mice). Healthy mice + normal saline (HMNC); Healthy mice + CNP 10 mg/kg (HM + CNP10); Healthy mice + CNP 20 mg/kg (HM + CNP20); Non-RA-infected mice (NRAI); RA-infected mice + Normal saline (RA + NC); RA-infected mice + PM 10 mg/kg (RA + PM 10); RA-infected mice + CNP 10 mg/kg (RA + CNP 10); RA-infected mice + CNP 20 mg/kg (RA + CNP 20); RA-infected mice + CNP 10 mg/kg + PM 5 mg/kg (RA + CNP 10+ PM5); RA-infected mice + CNP 20 mg/kg + PM 5 mg/kg (RA + CNP 20+ PM5). *P < 0.05 and ***P < 0.001 significant difference compared to HMNC; + *P* < 0.05, ++ *P* < 0.01, and +++ *P* < 0.001 compared to the RA + NC. & *P* < 0.05 compared to the RA + PM10.

In healthy mice (HMNC), liver function markers namely AST (106.3 U/L), ALT (64.2 U/L), and ALP (88.1 U/L)—as well as kidney function indicators BUN (32.4 mg/dL) and Cr (0.42 mg/dL) were observed to be within normal physiological ranges (Fig. 7). Administration of CNP at doses of 10–20 mg/kg resulted in a slight, non-significant elevation in these parameters (*P* > 0.05). Conversely, in the RA + NC group, which experienced reactivated toxoplasmosis, there was a marked and statistically significant increase in liver enzymes (AST: 189.5 U/L, ALT: 156.6 U/L, ALP: 173.8 U/L) and kidney markers (BUN: 57.2 mg/dL, Cr: 0.93 mg/dL) (*P* < 0.001), indicative of pronounced hepatic and renal injury. Treatment with either pyrimethamine alone (RA + PM10) or CNP alone (RA + CNP10/20) significantly ameliorated these biochemical indices relative to the RA + NC group (*P* < 0.001). Notably, combined therapy with CNP and pyrimethamine (RA + CNP20 + PM5) exerted a more pronounced effect, with all measured parameters approaching normal levels, suggesting a synergistic interaction in mitigating liver and kidney damage associated with reactivated toxoplasmosis. Numerous studies have demonstrated that CNPs synthesized via green methods exhibit reduced hepatotoxicity and nephrotoxicity compared to those produced through chemical synthesis, a difference attributed to the presence of natural surface agents derived from plant extracts (12, 13, 34). For instance, Khatami et al. (34) conducted an investigation using BALB/c mice and reported that CNP synthesized with Capparis spinosa extract, administered at doses up to 5 mg/kg, did not induce significant alterations in liver enzyme levels (AST, ALT, and ALP) or hematological parameters (*P* > 0.05), thereby supporting their biocompatibility. Similarly, Albalawi et al. (35) found that green-synthesized CNP did not elicit notable toxicity in hepatic or renal functions within a cutaneous leishmaniasis model while also demonstrating antiparasitic efficacy. Nonetheless, the potential protective mechanisms underlying these observations warrant further comprehensive investigation. Even though CNPs have shown promising therapeutic potential, their safety profile remains an important consideration for biomedical applications. Previous studies have indicated that metal-based nanoparticles may induce oxidative stress or cytotoxic effects depending on factors such as particle size, surface characteristics, and administered dose (34). In the present study, the evaluated biochemical and oxidative stress markers did not indicate severe adverse effects associated with CNP treatment, and some antioxidant parameters were improved in treated animals. However, further investigations are required to comprehensively evaluate the long-term safety of CNP, including detailed toxicological assessments, histopathological analyses, and pharmacokinetic studies to better understand their biodistribution and potential accumulation in tissues. Nanoparticle-based formulations may have particular clinical relevance for immunocompromised patients, who are highly susceptible to severe parasitic infections and often respond inadequately to conventional therapies. By improving drug delivery, bioavailability, and controlled release, nanoparticle systems may enhance therapeutic efficacy while potentially reducing drug-related toxicity. Although our findings are based on an experimental model, they suggest that nanoparticle-assisted strategies could represent promising adjunct approaches for improving the management of parasitic infections in immunocompromised populations, pending further pharmacological and clinical validation.

### Conclusion

The findings of the present study demonstrate that the administration of CNP in conjunction with a conventional pharmacological agent (PM) in a reactivated toxoplasmosis model resulted in a significant reduction of parasitic load across various tissues. Concurrently, there was an observed elevation in antioxidant enzyme activities, specifically SOD and GPx, alongside a decrease in MDA levels, collectively indicating a mitigation of oxidative stress and enhanced cellular protection. Furthermore, this combinatorial treatment augmented the expression of key immune cytokines, including TNF-α, NF-κB, and TLR4, suggesting an upregulation of innate immune responses and reinforcement of host defense mechanisms. These results underscore a multifaceted synergistic effect encompassing parasite proliferation control, oxidative stress modulation, and immune response enhancement, thereby highlighting the potential clinical utility of CNP as an adjunctive or alternative therapeutic strategy in the management of active toxoplasmosis. Future research endeavors should aim to elucidate the precise molecular mechanisms involved, conduct comprehensive safety evaluations over extended periods, and assess the translational applicability of these findings in preclinical and clinical settings. Such studies may facilitate the advancement of innovative nanoparticle-based therapeutics that concurrently exhibit antiparasitic, antioxidant, and immunomodulatory properties.

## Data Availability

Data are provided within the manuscript or Supplemental material.

## SUPPLEMENTAL MATERIAL

Supplemental material aac.00019-26-s0001.docx Fig. S1 to S3.

ASM does not own the copyrights to Supplemental Material that may be linked to, or accessed through, an article. The authors have granted ASM a non-exclusive, world-wide license to publish the Supplemental Material files. Please contact the corresponding author directly for reuse.
