# Supplementary material for: Targeting reactivated toxoplasmosis: therapeutic efficacy of the green-synthesized copper nanoparticles combined with pyrimethamine
Source: Antimicrob Agents Chemother. 2026 Jun 4;70(7):e00019-26. doi: 10.1128/aac.00019-26 (PMC13321808; doi:10.1128/aac.00019-26)
Supplement: Supplemental material — Fig. S1 to S3. [file aac.00019-26-s0001.docx]

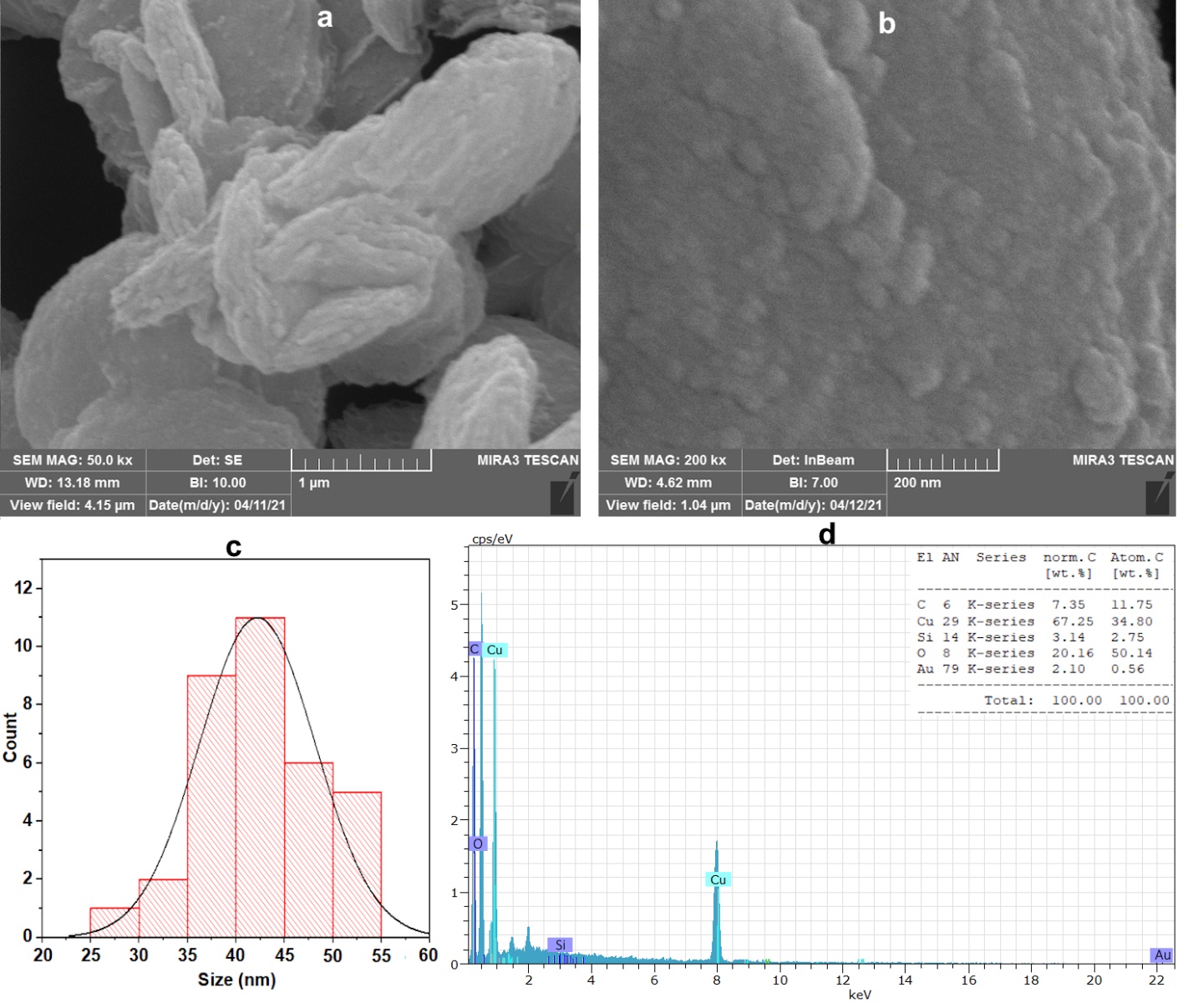


**Supplementary Fig. S1.** FESEM analysis (a and b), size distribution (c), and EDX analysis of green synthesized copper nanoparticles.


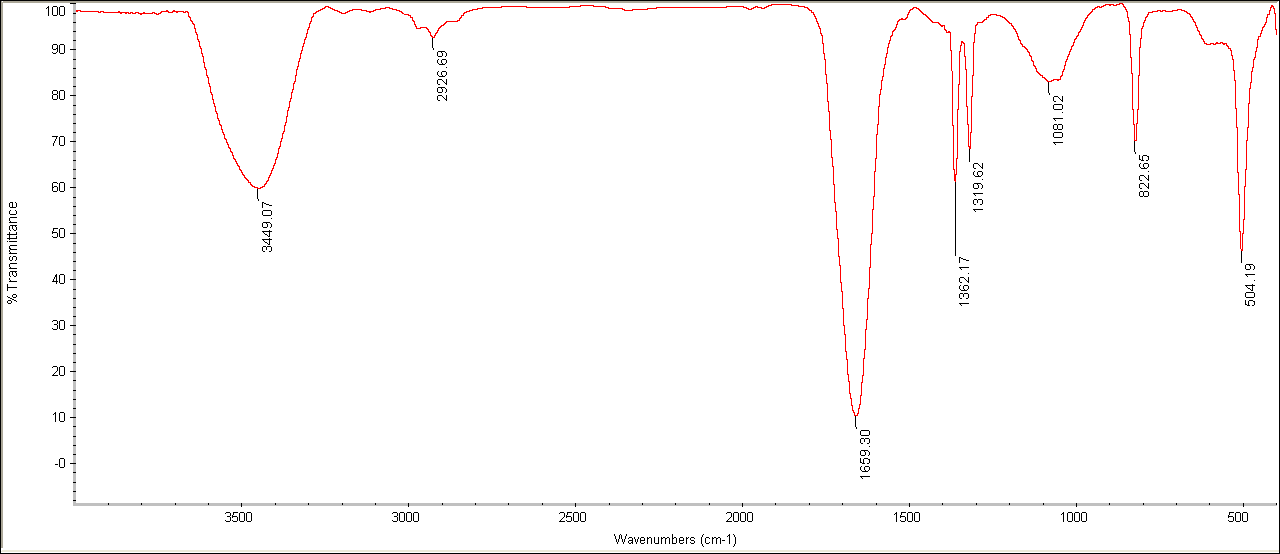


**Supplementary Fig. S2.** FTIR analysis of green synthesized copper nanoparticles.


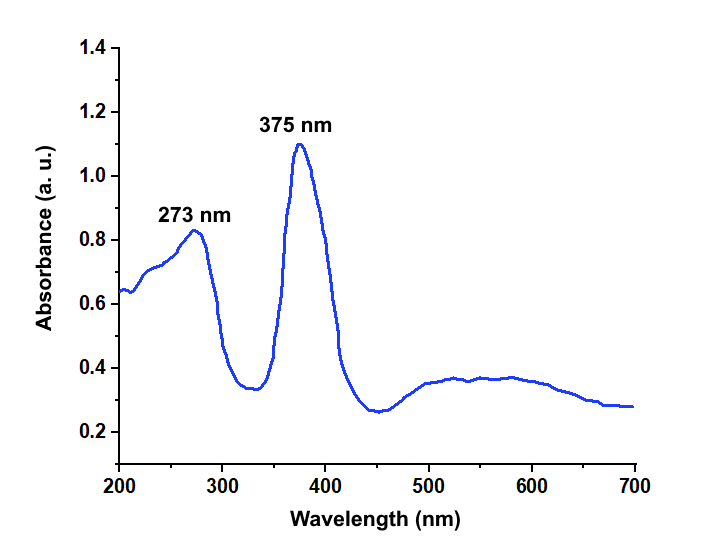


**Supplementary Fig. S3.** UV–visible absorption analysis of green synthesized copper nanoparticles.
